# Giant pulmonary artery aneurysm in a patient with vasoreactive pulmonary hypertension: a case report

**DOI:** 10.1186/1471-2261-11-64

**Published:** 2011-10-21

**Authors:** Inês Araújo, Pilar Escribano, María Jesus Lopez-Gude, Carmen Jimenez Lopez-Guarch, Maria Antonia Sanchez, Maria J Ruiz-Cano, Juan Delgado, Jose Cortina

**Affiliations:** 1 Hospital S. Francisco Xavier - Centro Hospitalar de Lisboa Ocidental, Internal Medicine Department, Lisboa, Portugal; 2 Hospital Universitario 12 de Octubre, Pulmonary Hypertension Unit, Madrid, Spain; 3 Hospital Universitario 12 de Octubre, Department of Cardiac Surgery, Madrid, Spain

## Abstract

**Background:**

Pulmonary artery aneurysms are a rare condition, frequently associated with pulmonary hypertension. However, the evolution and treatment of this pathology is still not clear.

**Case Presentation:**

The authors report a case of a 65-year old patient with pulmonary artery aneurysm associated with pulmonary arterial hypertension. Due to a positive vasoreactivity test, treatment with calcium channel blockers was started with near normalization of the right cardiac pressures. Nevertheless, after 20 months of treatment, the pulmonary artery aneurysm size remained unchanged with an associated severe pulmonary regurgitation and causing extrinsic compression of the main left coronary artery. Surgical correction was successfully performed.

**Conclusions:**

This is the first case report of a pulmonary artery aneurysm described to be associated with vasoreactive pulmonary hypertension in a living patient. Although medical therapy for pulmonary hypertension was started, surgical correction of the aneurysm was executed in order to prevent its future complications.

## Background

Pulmonary artery aneurysm refers to dilatation of the Pulmonary Artery (PA). Although some authors have proposed a cut-off of 4 cm in diameter in the past [1], a clear definition of PA aneurysm is not available as for aortic aneurysms [2].

It used to be an autopsy finding due to its asymptomatic course in the majority of the cases [3].

Several etiologies have been described in the pathogenesis of PA aneurysm, namely, pulmonary hypertension (PH), congenital heart disease, Behçet disease, infections such as the formerly prevalent syphilis, arteriovenous fistulas, connective tissue diseases, atherosclerosis and trauma [3,4]. Since the introduction of antibiotics non-infective causes of PA aneurysms have become more common [4].

With the technological advances in medicine, PA aneurysms have been diagnosed in living patients by computed tomography and magnetic resonance imaging [5].

The pathophysiology of the PA aneurysm is related to vessel wall stress that leads to vessel progressive dilatation or even rupture. Thus, once formed, an aneurysm tends to gradually enlarge [6].

Aneurysm dissection is a potentially fatal complication in about one third of the patients [6], but other serious complications of large PA aneurysms can also occur, namely, airway compression and thrombus formation on the PA [3]. Nonetheless, due to the low prevalence of PA aneurysms the optimal management is still unclear.

## Case Presentation

**Figure 1:**
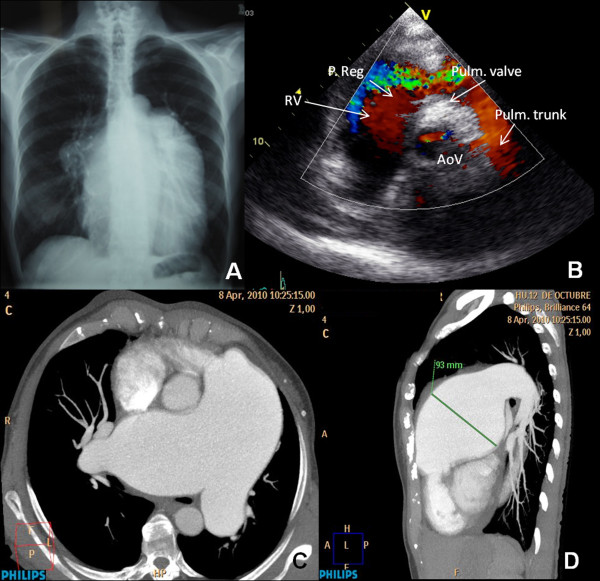
**Imaging exams displaying the pulmonary aneurysm and associated features.** A) Chest radiograph (posteroanterior view) shows cardiomegaly, dilated main pulmonary artery and right pulmonary artery. B) Ecocardiography: paraesternal view, at great vessels level. Color-Doppler shows severe pulmonary regurgitation. C) Multislice contrast computed tomographic scan of the thorax on axial projection shows a close contact of the main pulmonary artery to the chest wall, dilated main pulmonary artery and both of its branches which have a fusiform morphology. D) On sagital projection the widest diameter of the aneurysm can be measured.

A 65-year old woman was referred in January of 2009 to our pulmonary hypertension unit. She presented with a six-month history of dyspnea on major exertion without dizziness, syncope or chest pain. Her prior medical evaluation revealed PH and a PA aneurysm. At examination the patient was found to have a palpable systolic impulse within the second left anterior intercostal space, a grade 3/6 systolic murmur and a 2/6 diastolic murmur at the lower and upper left sternal border, respectively. Her chest radiography showed cardiomegaly and dilation of the main pulmonary artery and its branches (Figure 1A).

Secondary causes for PH were excluded according to the protocol described in the PH guidelines of the European Society of Cardiology [7]. A transthoracic echocardiography demonstrated a dilated right atrium and a dilated, hypertrophied right ventricle with severe systolic dysfunction (TAPSE 13), mild tricuspid regurgitation with PH (pulmonary artery systolic pressure, 80 mmHg), an aneurysmally dilated main PA (76 mm) and right (56 mm) and left (35 mm) PA branches and severe pulmonary regurgitation. The left sided-chambers were normal. A posterior and lateral moderate pericardium effusion could be seen. NT-proBNP was 1746 pg/mL.

A right cardiac catheterization showed a mean PA pressure of 61 mmHg with a cardiac output of 3.2 L/min. After vasoreactivity testing with intravenous epoprostenol, carried out on April of 2009, mean PA pressure dropped to 39 mmHg and concomitantly the cardiac output increased to 4.7 L/min. Calcium channel blockers were initiated, with excellent hemodynamic response and normalization of NT-proBNP values after five months of treatment.

The control right cardiac catheterization after 20 months of follow-up showed a mean PA pressure of 26 mmHg with a cardiac output of 5.54 L/min. Regardless of the near normalization of right cardiac pressures and the improvement of right ventricle function the patient maintained a severe pulmonary regurgitation (Figure 1B) and dilation of main PA and its branches on transthoracic echocardiography.

In order to evaluate the pulmonary vasculature the patient underwent a computed tomographic angiography showing an aneurysm affecting the main PA (65 mm on axial plane and 93 mm on sagital plane) and bilateral branches (right 41 mm, left 36 mm) (Figure 1C, D). A coronary angiography was carried out demonstrating an extrinsic compression of the left main coronary artery in relation to the PA aneurysm.

**Figure 2:**
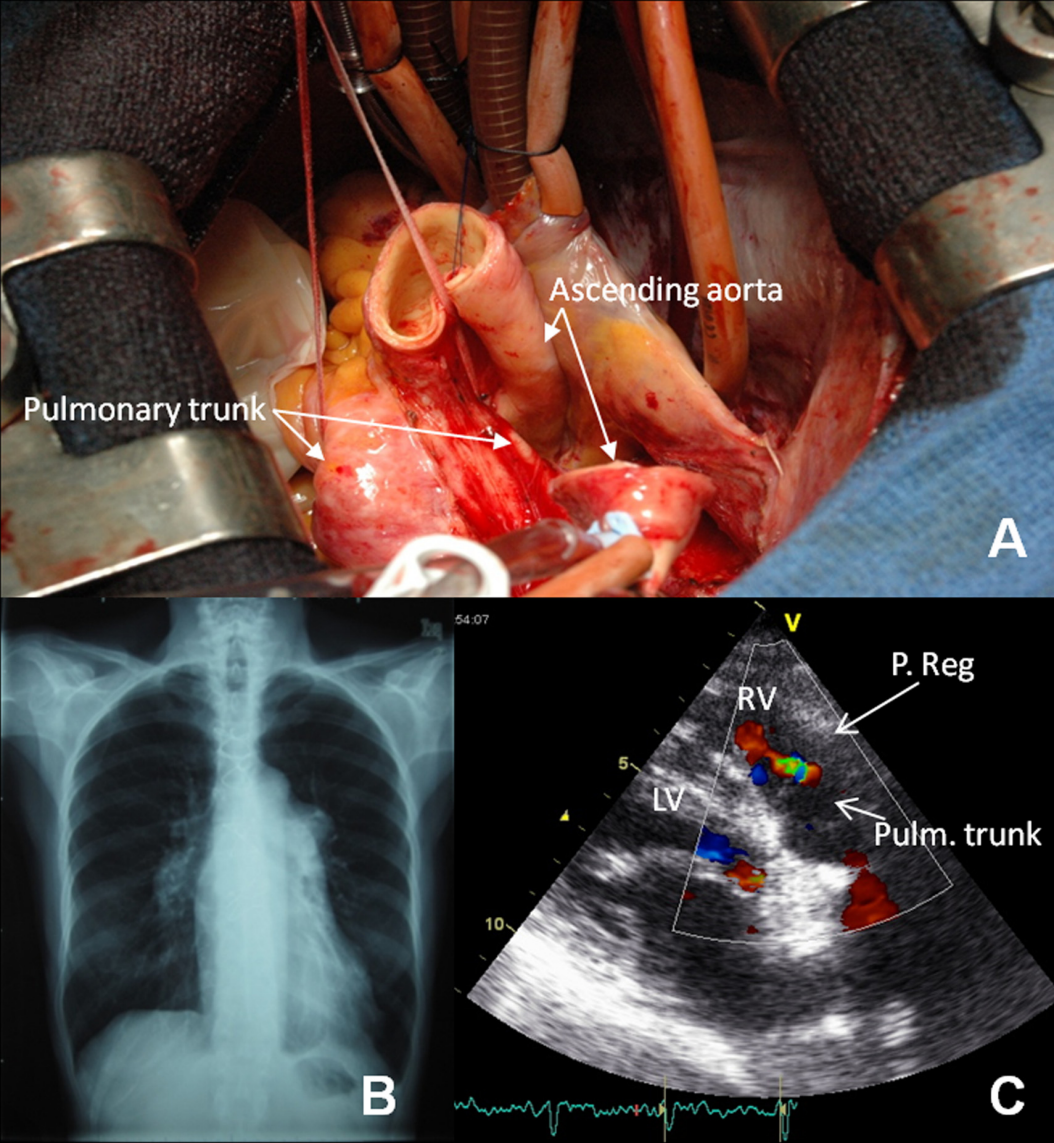
**Surgical procedure and imaging exams after corrective surgery.** A) Pulmonary artery aneurysm surgery: transverse aortotomy and visualization of the dilated pulmonary trunk. B) Post surgical chest radiograph (posteroanterior view) showing a narrower cardiac silhouette, without visualization of the main pulmonary artery. C) Post surgical echocardiogram showing only mild pulmonary regurgitation.

Surgery to correct the aneurysm was performed on December of 2010, with resection of the main PA and its two branches and their substitution by allograft tissue (Figure 2). There was no PH crisis after the surgery and also no residual pulmonary regurgitation. The patient has had an uneventful postoperative recovery, being asymptomatic and in NYHA class I.

In our patient, the PA aneurysm was associated with PH, this being the most prevalent association, described in 66% of the cases [3]. Diagnosis of the PA aneurysm was done during the investigation of PH, as no specific symptoms were present except for dyspnea that occurs in both PH and PA aneurysm. Other symptoms that may be present in patients with PA aneurysms are hemoptysis, cyanosis, clubbing, chest pain and cough [4]. However, none of these were present in our patient. Imaging such as computed tomography, echocardiography and coronary catheterization aided on the diagnosis and establishment of complications.

Intensive medical treatment has been one possible approach for PA aneurysms with clinical improvement of signs and symptoms [4,8,9] and so treatment for PH was started. However, despite being responsive to therapy with calcium channel blockers and near normalization of pulmonary vascular resistance, the aneurysm showed no size reduction after 20 months of treatment, suggesting that PH and PA aneurysms progress independently. Boerrigter *et al *have described that the normally observed progressive dilatation of PA is independent of hemodynamic changes in PH, such as, PA pressure and cardiac output. Rather, it can be related to changes on the vessel wall [10].

Although other authors have reported such giant PA aneurysms [3], this is the first described in a patient with vasoreactive severe PH. Moreover, it would be challenging to ascertain whether pulmonary regurgitation was the cause of pulmonary dilation or on the contrary, if it was a consequence of the progressive dilation of the pulmonary trunk; severe pulmonary regurgitation with volume overload might play a role on the persistence of the pulmonary aneurysm.

Surgical correction has also been described [11]. In fact, there are no clear guidelines on PA aneurysm treatment and patients have been handled individually. Surgery does not always have a formal indication, but as complications were already present, the authors decided to submit the patient to surgical correction. Coronary stenting could have been a potentially less invasive option to address the main left coronary artery compression. However, pulmonary artery surgery also corrects the severe pulmonary regurgitation and reduces the risk of rupture that was nonetheless present in our patient, due to the width of the aneurysm (over 9 cm). Currently, the patient has had an uneventful 10-month period follow-up.

## Conclusions

The case report presented illustrates a case of PA aneurysm diagnosed on a living patient with PH. Although this association is rather frequent, this is the first case described associated with vasoreactive PH. Despite medical therapy for PH being commenced no size reduction was observed on PA aneurysm. Aneurysmectomy was then performed and PA aneurysm was successfully replaced by allograft tissue.

## Consent

Written informed consent was obtained from the patient for publication of this case report and any accompanying images. A copy of the written consent is available for review by the Editor-in-Chief of this journal.

## Abbreviations

NYHA: New York Heart Association; PA: pulmonary artery; PH: pulmonary hypertension; TAPSE: tricuspid annular plane systolic excursion.

## Competing interests

The authors declare that they have no competing interests.

## Authors' contributions

IA and PE have done the patient's follow-up and drafted the manuscript. MJLG and JC have done the surgery and provided the photographs of the surgery. CJLG and MAS have documented the aneurysm by echocardiography and computed tomography, have done the imagiological follow-up and have provided the respective photographs. MJRC and JD have helped on the manuscript drafting and revision. All authors read and approved the final manuscript.

## Pre-publication history

The pre-publication history for this paper can be accessed here:

http://www.biomedcentral.com/1471-2261/11/64/prepub
